# Uterine Angioleiomyoma: Clinical and Histopathologic Differentiation of an Underrecognized Mimicker of Uterine Leiomyoma

**DOI:** 10.1177/10668969241256117

**Published:** 2024-07-25

**Authors:** Tuan Pham, Joshua M. Peterson, Hasanain Hasan, Mariangela Gomez

**Affiliations:** 1 John Sealy School of Medicine, University of Texas Medical Branch, Galveston, TX, USA; 2 Department of Pathology, University of Texas Medical Branch, Galveston, TX, USA

**Keywords:** angioleiomyoma, uterine angioleiomyoma, leiomyoma, leiomyoma variants, abnormal uterine bleeding, myometrium, myopericytoma, PEComa, fibroid

## Abstract

Angioleiomyoma is an uncommon benign neoplasm of mesenchymal origin that arises from perivascular smooth muscle cells. This soft tissue neoplasm usually occurs in the dermal or subcutaneous tissues of the extremities, head and neck, or trunk with fewer than 40 reported angioleiomyomas arising in the uterine corpus. Herein we report a uterine angioleiomyoma in a 44-year-old G5P4 Hispanic woman with a longstanding history of recurrent abdominal pain, pelvic organ prolapse, abnormal uterine bleeding, anemia, and hypertension. The patient underwent surgical treatment with total laparoscopic hysterectomy with bilateral salpingectomy and a uterosacral ligament suspension. Uterine angioleiomyoma was diagnosed post-operatively based on gross and microscopic features. The location of the uterine angioleiomyoma within the myometrium corresponded with contrast enhancement apparent on preoperative imaging. This and other uterine angioleiomyomas have characteristic imaging, macroscopic, and microscopic features which distinguish it from leiomyoma. Enhancing awareness of this underrecognized entity will facilitate precise diagnosis and thereby enable improved understanding of the clinicopathological characteristics of uterine angioleiomyoma.

## Introduction

Angioleiomyoma is an uncommon benign neoplasm of mesenchymal origin that arises from perivascular smooth muscle cells and grows concentrically in a fascicular pattern around vascular channels.^1^ Whereas angioleiomyoma most commonly presents as a palpable nodule in the dermal or subcutaneous tissue of the extremities (and relatively less so in the head, neck, and trunk), uterine angioleiomyoma is an uncommon location for this rare soft tissue neoplasm.^2,3^ A 2016 review found the incidence of uterine angioleiomyoma among 2270 hysterectomy and myomectomy specimens for uterine fibroid to be between 0.34% and 0.40%, and a 2019 systematic review found only 34 such tumors reported in the English literature.^4,5^ Garg et al describe that uterine angioleiomyoma occurs more commonly in middle-aged women with a typical age range from 30 to 69 years old.^3^ Clinical manifestations include abnormal uterine bleeding, lower abdominal mass, and abdominal pain with some patients also presenting with anemia and hypertension.^3,5^ In this report, we present a uterine angioleiomyoma detected in a 44-year-old woman with a longstanding history of recurrent abdominal pain, abnormal uterine bleeding, pelvic organ prolapse, hypertension, and anemia, and discuss its salient distinguishing factors from leiomyoma and other similar mesenchymal tumors such as myopericytoma, PEComa, and endometrial stromal tumors.

## Report

**Figure 1.**
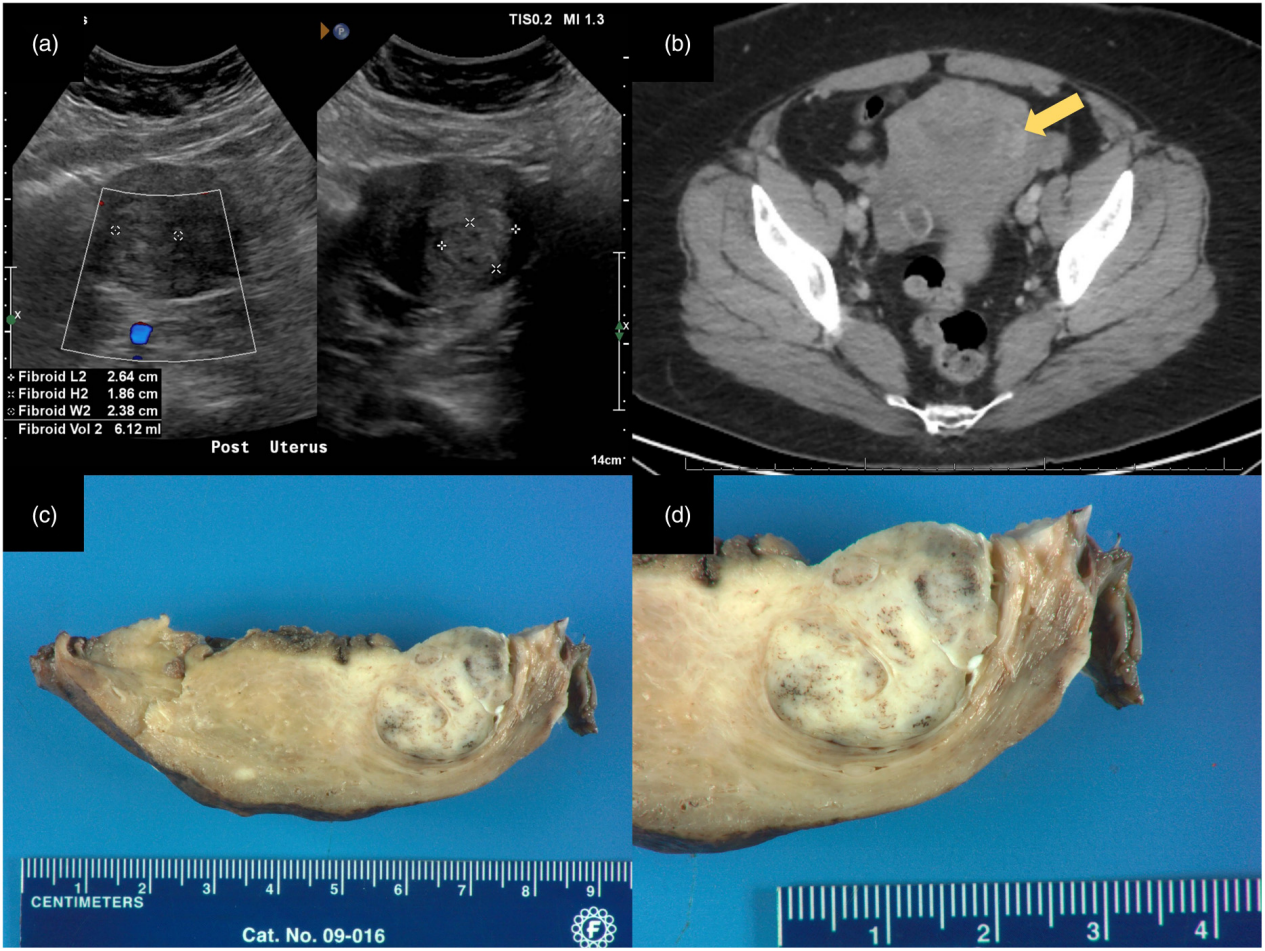
(a) Posterior uterine ultrasonogram showing a hyperechoic lesion in the posterior left uterine wall which measured 2.6 × 1.9 × 2.4 cm^3^; (b) CT abdomen/pelvis with contrast demonstrating enhancing lesion of posterior left uterine wall (yellow arrow; axial view); (c, d) Gross photographs of uterine angioleiomyoma showing a well-circumscribed mixed tan and blue-grey mass at the left posterior lateral myometrium with pinpoint hemorrhages and variegated surface. CT, computed tomography.

A 44-year-old G5P4 Hispanic woman with chronic anemia and essential hypertension was being followed by her gynecologists for menorrhagia, dysmenorrhea, and a bulging sensation of in her pelvis that began approximately 1.5 years earlier. She also experienced stress incontinence related to this bulging sensation. She reported worsening menorrhagia beginning 5.5 years earlier which was associated with worsening dysmenorrhea. During her initial work-up for abnormal uterine bleeding, uterine prolapse was found by Valsalva maneuver. Despite ongoing ferrous sulfate supplementation, her hemoglobin levels progressively declined to a nadir of 9.9 g/dL. Pelvic ultrasound demonstrated an enlarged uterus measuring 10.7 × 5.8 × 7.7 cm^3^ and the presence of three uterine fibroids, the largest measuring 3.9 × 2.2 × 3.2 cm^3^ located on the posterior uterine wall (Figure 1a). Computed tomography (CT) imaging with contrast corroborated these findings and demonstrated contrast enhancement of one intermediate-sized fibroid of the left posterior lateral myometrium (Figure 1b) with the absence of contrast enhancement in the other fibroids. From these findings, she was diagnosed with abnormal uterine bleeding leiomyoma type and pelvic organ prolapse. Pap smear showed atypical squamous cells of undetermined significance, with subsequent colposcopic biopsy demonstrating no histopathologic abnormalities. Following complete preoperative work-up, total laparoscopic hysterectomy with bilateral salpingectomy and a uterosacral ligament suspension procedure were performed without complication.

**Figure 2.**
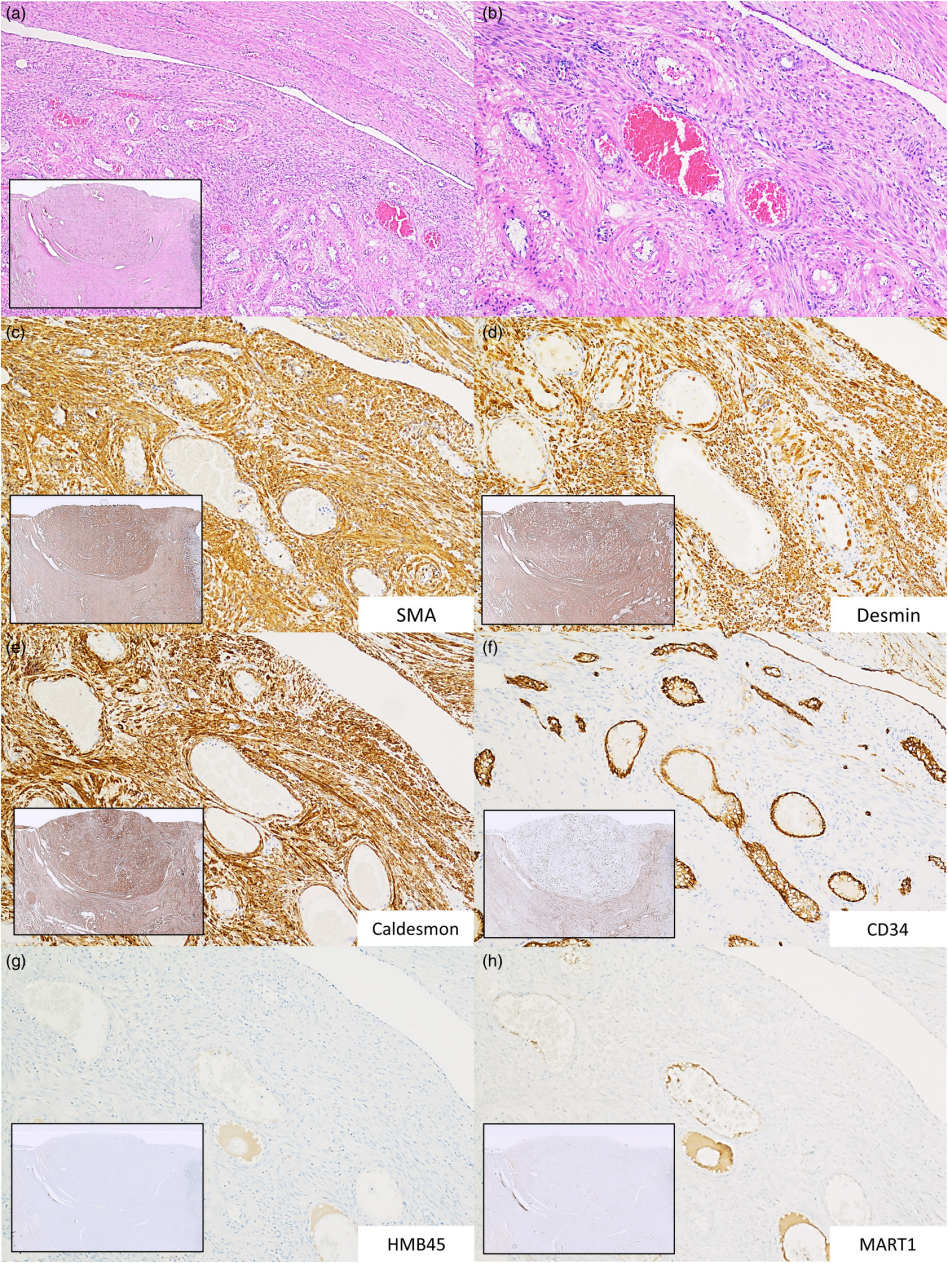
Photomicrographs of H&E and IHC staining of the interface of the resected uterine angioleiomyoma (U-ALM) with adjacent normal myometrium (a, b) illustrating perivascular smooth muscle fascicles surrounding thickened blood vessels (H&E, 5× (inset), 40×, and 100× mag.); (c) SMA, diffuse positive staining in tumor and myometrium (5× (inset) and 100× mag.); (d) Desmin, diffuse positive staining in tumor and myometrium (5× (inset) and 100× mag.); (e) Caldesmon, diffuse positive staining in tumor and myometrium (5× (inset) and 100× mag.); (f) CD34, focal staining of endothelial cells only within tumor in contrast to patchy positive staining of myometrial stroma (5× (inset) and 100× mag.); (g) HMB-45, diffuse negative staining in tumor and myometrium (5× (inset) and 100× mag.); and (h) Melan-A, diffuse negative staining in tumor and myometrium (5× (inset) and 100× mag.). IHC, immunohistochemical.

On gross examination, the uterus measured 12.7 × 8.8 × 6.4 cm^3^ and weighed 293 grams. Macroscopic examination revealed a myometrium with multiple firm well-circumscribed tan-white leiomyomas, the largest (2.7 cm) protruding into the endometrial canal. However, the cut surface of one well-circumscribed lesion within the left posterior lateral myometrium (1.7 cm) was distinct from adjacent lesions due to its soft, deformable texture, pinpoint hemorrhages, and mixed tan and blue-grey stroma. Microscopic examination of this mass showed prominent thick-walled blood vessels interlaced by well-differentiated smooth muscle fascicles (Figure 2a and b). No atypia, pleomorphism, mitosis, or necrosis was identified. Immunohistochemical (IHC) staining for SMA, desmin (*DES*), and caldesmon (*CALD1*) resulted positive, demonstrating the smooth muscle lineage of the tumor, whereas CD34 highlighted only the vascular component (Figure 2c-f). Staining for HMB45 (*PMEL*) and MART1 (*MLANA*) returned negative (Figure 2g and h). Histologic findings and staining pattern established a diagnosis of uterine angioleiomyoma, venous type. At clinical follow–up, the patient remained in stable condition with no apparent recurrence.

## Discussion

Uterine leiomyomas arise from smooth muscle cells of the myometrium and are the most common benign neoplasms in women of reproductive age.^6^ Angioleiomyoma, however, arises from perivascular smooth muscle cells and is recognized by the World Health Organization (WHO) as a distinct soft tissue neoplasm rather than a variant of uterine leiomyoma.^2^ Although the etiology of angioleiomyoma remains unclear, it is most commonly found in the dermis and subcutaneous tissues of extremities, trunk, head, and neck. Embryologically, sub-endometrial myometrium originates from the Müllerian duct, whereas sub-serosal myometrium arises from the mesenchyme.^7^ While there is some ongoing speculation regarding which precursor cell population gives rise to uterine angioleiomyoma specifically, the presence of both perivascular smooth muscle cells and non-Müllerian mesenchymal smooth muscle in the uterine corpus together with the relative rarity of development of angioleiomyoma in the uterus suggests a common precursor lineage with extra-uterine soft tissue angioleiomyoma.^8^

Preoperative diagnosis of uterine angioleiomyoma is hindered by its nonspecific appearance on routine imaging and shared clinical features with uterine leiomyomata.^4,9^ In soft tissues, imaging features of angioleiomyoma which aid distinction from leiomyoma include hypervascularity and feeder vessel identification on color Doppler examination. Distinguishing CT features include tissue attenuation similar to skeletal muscle and enhancement with contrast. The patient presented here is illustrative, as contrast enhancement was noted in the myometrial mass where the uterine angioleiomyoma was identified, but not in the other masses which were subsequently diagnosed as leiomyomas (Figure 1b). The lesion is most accurately identified by MRI, where it is isointense or hypertense to skeletal muscle on T1-weighted images with a peripheral rim of attenuated signal on T1- and T2-weighted images with a “dark reticular sign” on T2-weighted images.^10^ However, the relevance of MRI is limited as this is not a routine preoperative imaging modality in a patient suspected of abnormal uterine bleeding, which is the most common and persistent problem for patients with both uterine leiomyomata and uterine angioleiomyoma.^4,5^ While the etiology remains unclear, proposals for the cause of heavy bleeding in uterine angioleiomyoma include hypertension as an underlying factor (as with our patient);^11^ others postulate that the mechanism is similar to the proposed mechanism for bleeding in leiomyoma: the disruption of angiogenesis by dysregulation of growth factors and/or their receptors leading to increased risk for spontaneous bleeding.^12^ Whereas speculation on the relationship of uterine angioleiomyoma to abnormal uterine bleeding in this patient is confounded by the presence of multiple coexisting leiomyomas, patients with uterine angioleiomyoma have been previously reported to become anemic over time, similar to our patient. Fortunately, uterine angioleiomyoma is a benign tumor with excellent prognosis after gynecologic surgery.^2,3^ There have been very seldom reported recurrences in patients treated by myomectomy and no observed malignant transformation.^4^

On gross macroscopic examination, uterine leiomyomas are circumscribed masses which are solid, rubbery, and firm with white-tan coloration and a whorled, bulging cut surfaces.^13^ Uterine angioleiomyoma often have a similar appearance but tend to have interspersed hemorrhagic regions giving a marbled appearance with spots of pink, brown, and grey as a result of their more prominent vascular components.^3,5,8^ However, these areas may not always be apparent in some uterine angioleiomyoma and likely vary with subtype, so it may be difficult to differentiate leiomyomas from uterine angioleiomyoma on gross appearance alone. On histological exam, leiomyoma are composed of long fascicles of smooth muscle cells in bland appearance with vascular networks described as being hyper-vascularized capsules with hypo-vascular cores, supplied in part via small peripheral arteries linking to arterial plexuses with few unevenly distributed intra-fibroid vessels.^3,8,14^ In contrast, uterine angioleiomyoma are also composed of interlacing fascicles of smooth muscle cells, but these tend to surround evenly distributed thick-walled muscular vasculature (in venous subtype) or thin-walled cavernous vessels (in cavernous subtype) which are prominent and present diffusely throughout the lesion.

This diffuse vascular network is highlighted as essential diagnostic criteria for angioleiomyoma, although the vascular channels can be of various sizes.^2^ Angioleiomyomas are subclassified by their histologic vascular morphology into three categories: solid, venous, and cavernous; they may also appear in mixed morphology and with minor histological variations such as adipocytic metaplasia or with hyalinization/calcification.^2^ The venous type is described as thickened muscle-encased blood vessels that are distinguishable from surrounding intervascular smooth muscle bundles, the cavernous type as stretched and dilated thin-walled vascular channels, and the solid type as compacted with fissure-like vascular channels.^2^ The venous subtype of angioleiomyoma (the subtype of the lesion presented herein) is both the less common if the subtypes and the most likely to be seen in the head and neck. These variant morphologies have no known clinical significance at this time.

The IHC profile of uterine angioleiomyoma can aid with the differential diagnosis of these tumors. Angioleiomyoma has been observed to show strong immunoreactivity and stains diffusely for SMA, calponin (*CNN1*), and caldesmon. Desmin IHC reportedly has variable expression in angioleiomyoma, but some studies observed strong positivity in uterine angioleiomyoma much like in the presented tumor (Figure 2d).^2,3,5^ There is also observed weak and focal staining for CD34 by endothelial cells within angioleiomyoma, and in contrast to other tumors of pericyte origin (eg, angiomyolipoma and PEComa), HMB45 and MART1 are negative (this was also observed in the presented tumor: Figure 2f-h). Of note, rare incidences of uterine leiomyoma with scant positive staining for HMB45 have been reported.^15–17^ However, we speculate these reports may represent typical staining patterns of lesions of pericyte lineage such as the monotypic (fat-poor) variant of angiomyolipoma, another rare mimicker of leiomyoma occurring in the uterus.^18^

Besides leiomyoma, other mesenchymal tumors occurring in the uterine corpus—such as PEComa, endometrial stromal tumors, and myopericytoma—display biphasic proliferations with prominent blood vessels and perivascular growth patterns, and uterine angioleiomyoma should be included on the differential diagnosis of these neoplasms. Salient examples of such neoplasms include PEComa, endometrial stromal tumors, and myopericytoma.^3,8^ PEComas are neoplasms with malignant potential of pericyte lineage composed of epithelioid cells commonly with clear to eosinophilic granular cytoplasm growing radially around adjacent vessels.^2,19^ Importantly, PEComas stain for melanocytic markers such as HMB45 and MART1, allowing them to be differentiated from other mesenchymal tumors such as uterine angioleiomyoma. Endometrial stromal tumors comprise a family of neoplastic proliferations of endometrial stromal cells, from the rare benign endometrial stromal nodule to the more common malignant low- and high-grade endometrial stromal sarcomas. Endometrial stromal tumors are composed of small cells with scarce cytoplasm and oval- to spindle-shaped nuclei resembling endometrial stromal cells with a whorled growth pattern encasing proliferative type arterioles with thickened walls, reminiscent of the venous subtype of angioleiomyoma.^18,20^ Sánchez-Iglesias et al remarked that their reported uterine angioleiomyoma was misdiagnosed as an endometrial stroma sarcoma when examined via frozen section.^5^ By IHC, endometrial stromal tumors are readily distinguished from uterine angioleiomyoma by positive staining for CD10 (*MME*) and SMA and negative staining for caldesmon.^20,21^ Myopericytoma has been described by the WHO as a perivascular myoid neoplasm on a morphological continuum with angioleiomyoma.^2^ Its appearance consists of bluish oval- or spindle-shaped cells arranged in a concentric pattern surrounding multiple small vessels with increased perivascular cellularity and, occasionally, hemangiopericytoma-like vessels (staghorn vessels). This stands in contrast the more fascicular architecture and overall greater eosinophilic appearance of angioleiomyoma.^22^ Both myopericytomas and angioleiomyoma cells stain diffusely positive for caldesmon and SMA, and myopericytomas are mostly negative for desmin with some observed rare focal positivity.^2,22^ In a case series of uterine myopericytomas, it was suggested that staining for CD10 aids in distinction of angioleiomyoma from myopericytoma, as CD10 is expected to be positive in angioleiomyoma and negative in myopericytoma.^23^ Other less common mesenchymal tumors to consider in the differential diagnosis include uterine tumor resembling ovarian sex cord tumor and inflammatory myofibroblast tumor; both are of rare occurrence and the diagnosis is mostly by exclusion of other common entities. Uterine tumor resembling ovarian sex cord tumor shows tumor cells with organized sheets, cords and nests that stains positive for calretinin (*CALB2*), WT1 and inhibin alpha (*INHA*) whereas inflammatory myofibroblast tumor stains positive for ALK.^24,25^ Although awareness of histological mimics is important for process diagnosis, priority should always be given to assessment for infiltrative growth, atypia, pleomorphism, mitoses, and necrosis which could warrant consideration of a malignant process (eg, leiomyosarcoma, endometrial stromal sarcoma).

## Conclusion

Uterine angioleiomyoma is an exceedingly rare benign soft tissue neoplasm of the perivascular smooth muscle cells occurring in the uterine myometrium which mimics leiomyoma clinically and is difficult to diagnose preoperatively. Retrospectively, CT contrast enhancement of uterine angioleiomyoma effectively distinguished this lesion from concurrent leiomyomas. Grossly, the neoplasm can be distinguished from leiomyoma by its marbled appearance with pinpoint hemorrhage and relatively soft texture on cut surface. It is distinguished histologically from leiomyoma by its abundant and evenly distributed vessels with a concentric and fascicular growth pattern. When necessary, IHC staining effectively differentiates this lesion from histopathologic mimics, including leiomyoma, PEComa, endometrial stromal tumors, and myopericytoma. Special attention should be given to atypia, pleomorphism, mitosis, and necrosis that could indicate a malignant process. Improving recognition of this likely underdiagnosed entity will enable accurate diagnosis and subsequent increased understanding of the clinicopathological features of uterine angioleiomyoma.
